# A Matched Case Control Study of Surgically and Non-surgically Treated Patent Ductus Arteriosus in Extremely Pre-term Infants

**DOI:** 10.3389/fped.2021.648372

**Published:** 2021-03-18

**Authors:** Karl Wilhelm Olsson, Sawin Youssef, Mattias Kjellberg, Renske Raaijmakers, Richard Sindelar

**Affiliations:** ^1^Department of Women's and Children's Health, Uppsala University, Uppsala, Sweden; ^2^Division of Neonatology, Department of Pediatrics, Sahlgrenska University Hospital, Gothenburg, Sweden

**Keywords:** case-control study, echocardiography, extreme pre-maturity, ligation, patent ductus arteriosus

## Abstract

**Introduction:** There are still uncertainties about the timing and indication for surgical ligation of patent ductus arteriosus (PDA) in pre-term infants, where lower gestational age (GA) usually is predictive for surgical treatment.

**Objective:** Our aim was to assess differences in clinical characteristics and outcomes between surgically treated and matched non-surgically treated PDA in extremely pre-term infants.

**Methods:** All extremely pre-term infants born 2010–2016 with surgically treated PDA (Ligated group; *n* = 44) were compared to non-surgically treated infants (Control group; *n* = 44) matched for gestational age (+/−1 week) and time of birth (+/−1 month). Perinatal parameters, echocardiographic variables, details of pharmacological PDA treatment, morbidity, and mortality were assessed.

**Result:** Mean GA and birthweight were similar between the Ligated group (24^+5^ ± 1^+3^ weeks and 668 ± 170 g) and the Control group (24^+5^ ± 1^+3^ weeks and 704 ± 166 g; *p* = 1.000 and *p* = 0.319, respectively). Infants in the Ligated group had larger ductal diameters prior to pharmacological treatment, and lack of diameter decrease and PDA closure after treatment (*p* = 0.022, *p* = 0.043 and 0.006, respectively). Transfusions, post-natal steroids and invasive respiratory support were more common in the Ligated group. Except for a higher incidence of severe bronchopulmonary dysplasia (BPD) in the Ligated group there were no other differences in outcomes or mortality between the groups.

**Conclusion:** Early large ductal diameter and reduced responsiveness to pharmacological treatment predicted the need for future surgical ligation in this matched cohort study of extremely pre-term infants where the effect of GA and differences in treatment strategies were excluded. Besides an increased incidence of severe BPD in the Ligated group, no other differences in morbidity or mortality were detected.

## Introduction

Patency of the ductus arteriosus in newborn pre-term infants continues to be one of the most challenging problems in neonatal intensive care units. Despite extensive research efforts, there are still uncertainties about the diagnostic criteria and treatment strategies for patent ductus arteriosus (PDA) in pre-term infants, and specifically in extremely pre-term newborn infants (1, 2).

Before pharmacological treatment was available, early surgical ligation was performed to close PDA in pre-term infants (3). As the initial reduction in morbidity after surgical closure did not translate into improved outcomes, pharmacological treatment with cyclooxygenase inhibitors or acetaminophen eventually became the first-hand choice for PDA treatment. Surgery was then reserved for infants with persistent hemodynamically significant PDA where either contraindications for pharmacological treatment were present or for infants that did not respond to pharmacological treatment (4–6). As it has been recognized that there is a high degree of spontaneous closure of ductus arteriosus and a low efficiency of pharmacological treatment, the application of pharmacological treatment has also declined during the last decade (7). Recently, arguments have also been presented in favor of conservative management of PDA with neither pharmacological nor surgical treatment in pre-term infants (8–10).

Never the less, PDA in extremely pre-term infants (<28 weeks gestational age) might lead to significant hemodynamic effects with cardiac and pulmonary complications such as heart failure, prolonged mechanical ventilation and increased incidence of bronchopulmonary dysplasia (BPD) (11). With improved survival of extremely pre-term infants, these infants have a higher risk of developing a symptomatic PDA that either exhibits contraindications for or fails to respond to pharmacological treatment, whereby the need for surgical treatment might increase in this cohort (12, 13).

There are few studies on surgically treated PDA in infants born before 28 weeks gestational age, and results from studies of infants born at a higher gestational age might be difficult to apply in extremely pre-term infants. Also, many studies on surgically treated PDA were conducted during an era where surfactant therapy, antenatal steroids, non-invasive positive pressure ventilation, routine echocardiography, or pharmacological treatment of PDA were not fully established (14, 15). The aim of this matched case control study of PDA was to investigate the differences in peri- and post-natal characteristics, pharmacological PDA treatment, and outcomes between surgically and non-surgically treated extremely pre-term infants. By matching surgically treated infants with infants born within the same month and week of GA, we wanted to exclude the possible effects of maturation and different treatment strategies over time, factors that could interfere with the studied parameters (16).

## Subjects and Methods

### Subjects

From January 2010 to December 2016, 463 infants were born at 22 + 0 to 27 + 6 weeks GA and admitted to the neonatal intensive care unit at Uppsala University Children's Hospital. Forty-four infants were surgically treated for PDA (Ligated group). Each infant in the Ligated group was matched to an infant in the Control group from the Swedish Neonatal Quality Register (44 out of 101 eligible infants), blindly as to any other outcomes except for (1) not having undergone surgery for PDA, (2) being born within +/−1 month of the birthdate, and (3) within +/−1 week of the gestational age of the infant in the Ligated group. Infants with congenital anomalies or heart conditions other than PDA or foramen ovale were excluded from both groups in this study. This retrospective observational study was approved by the Regional Ethical Review Board, D:nr 933-17.

### Perinatal Parameters, Post-Natal Treatments, and Outcomes

Medical records were assessed for perinatal parameters: birthweight, sex, prolonged pre-mature rupture of membranes, administration of pre-natal steroids, mode of delivery, and Apgar-scores at 1, 5 and 10 min. Information on number and volume of erythrocyte transfusions, administration of inotropic drugs, post-natal steroid administration, pharmacological PDA treatment, and surgical treatment of PDA were collected. Clinical outcomes data were assessed for BPD [graded mild, moderate or severe according to Higgins et al. (17)], intraventricular hemorrhage (IVH; graded according to Papile's criteria) (18), retinopathy of pre-maturity (ROP; graded according to the International Classification of ROP) (19), necrotizing enterocolitis (NEC; graded according to the modified Bell's criteria) (20), sepsis (defined as positive blood culture with clinical signs of infection; or as negative blood culture with clinical signs of infection and positive laboratory infectious tests), and mortality.

### Ventilatory Parameters

The patient data management/monitoring systems IntelliVue Clinical Information Portfolio (ICIP; Philips Healthcare, Eindhoven, Netherlands) and MetaVision (Metavision; iMDsoft, Düsseldorf, Germany) were used at the unit during the studied period. Data obtained from these monitoring systems included total days with ventilatory support, mode of ventilatory support, ventilator settings, and supplementary oxygen administration.

### Echocardiography

Infants in the unit routinely underwent echocardiographic examinations during the first day of life with additional examinations if they exhibited clinical signs of PDA. Examinations were also performed within 24 h of treatment completion, if the infant's clinical condition called for it, and before discharge. The initial assessment was carried out by a pediatric cardiologist and the following examinations either by a pediatric cardiologist or neonatologist with appropriate training and experience (21).

Examinations included assessment of the ductal diameter measured at the narrowest point from the parasternal short axis view, ductal flow velocity measured in line with the ductal flow from the parasternal short axis view, left atrium to aortic root ratio measured from the parasternal long axis and flow in the descending aorta assessed above and below the ductal orifice (22). Ductal closure was defined as absence of identifiable flow in the ductus arteriosus by color Doppler.

### Pharmacological Treatment of PDA

Hemodynamically significant PDA (hsPDA) was defined as an open ductus arteriosus with predominantly left-to-right flow after the first day of life and either: (A) ductal diameter of ≥1.5 mm; (B) LA/Ao of >1.5; or (C) an appropriate view of the descending aorta showing absent or reversed flow during diastole (22). Pharmacological treatment was initiated if hsPDA was identified before the seventh day of life. Contraindications were renal failure (serum creatinine >120 μmol/L or serum urea >12 mmol/L), thrombocytopenia (platelets <50 × 10^9^/L), recent IVH grade II–IV, and NEC. Three doses of Ibuprofen (5 mg/mL, Pedea, Orphan Europe Nordic, Stockholm, Sweden) were administered intravenously with an initial dose of 10 mg/kg infused over 20 min and subsequent doses of 5 mg/kg at 24 and 48 h after the initial dose. No additional pharmacological ductus treatment was administered after the first course.

### Surgical Treatment of PDA

Surgical ligation was carried out if pharmacological treatment was contraindicated or failed and hsPDA still persisted in combination with clinical signs of deterioration in pulmonary and systemic circulation, i.e. clinically hemodynamically significant; or if PDA reopened and was found to be clinically hemodynamically significant after pharmacological treatment (23).

### Statistical Analysis

Matlab (The Mathworks Inc., Natick, Massachusetts) was used for statistical analysis. Categorical variables were statistically analyzed using Fisher exact test. Continuous variables were analyzed using Student's *t*-test or Mann–Whitney *U*-test. Normal distribution of parameters was tested by Kolmogorov-Smirnov's goodness of fit test. Categorical variables are presented as number and percent, and continuous variables are presented as mean and standard deviation (SD) or median and interquartile range (IQR). All *p*-values presented are two-tailed, and a *p* < 0.05 was considered significant. Multivariate logistic regression was used to examine the relationship between treatments possibly affecting ductal closure (post-natal steroids, transfusions, pharmacological PDA treatment, and ventilatory days), ductal patency/diameter on first examination, and subsequent surgical ligation.

## Results

There was no difference in GA or birthweight between the Ligated group and the Control group. Perinatal characteristics for all infants are presented in Table 1. Of all infants born at 22–27 weeks GA during the study period, only 9.5% were surgically ligated. Infants in the Ligated group had surgery for PDA at a mean post-natal age of 50 ± 26 days.

**Table 1:** Perinatal characteristics and treatments.

	**Ligated**	**Control**	
	***n* = 44**	***n* = 44**	***p***
**Perinatal characteristics**			
Gestational age, weeks	24^+5^ ± 1^+3^	24^+5^ ± 1^+3^	1.000
Birthweight, grams	668 ± 170	704 ± 166	0.319
Male, *n*	25 (57%)	21 (48%)	0.522
Pre-term pre-mature rupture of membranes, *n*	11 (25%)	12 (27%)	1.000
Pre-natal steroids, *n*	44 (100%)	44 (100%)	1.000
Cesarean section, *n*	26 (59%)	22 (50%)	0.521
Apgar 1	4.0 ± 2.2	5.2 ± 2.4	0.018
Apgar 5	5.8 ± 2.2	6.6 ± 2.0	0.064
Apgar 10	7.4 ± 2.1	8.0 ± 1.5	0.166
**Treatment**			
Transfusions, *n*	5 (3–6)	3 (1–4)	<0.001
Transfusion volume, ml/kg	75 (45–90)	45 (15–60)	<0.001
Inotropic support, *n*	10 (23%)	6 (14%)	0.408
Post-natal steroids, *n*	11 (25%)	1 (2%)	0.004
Invasive ventilation, days	47 (38–68)	20 (3–36)	<0.001
CPAP, days	14 (8–26)	13 (5–32)	0.729
Invasive and non-invasive ventilation, days	63 (49–99)	40 (13–70)	<0.001
Pharmacological PDA-treatment, *n*	23 (52%)	22 (50%)	1.000
Time of pharmacological treatment, days	5.2 ± 4.7	4.4 ± 6.6	0.654

*Continuous variables are presented as mean ± standard deviation or median and interquartile range (IQR), and categorical variables are presented as number and percent. CPAP, Continuous positive airway pressure; PDA, Patent ductus arteriosus*.

### Perinatal Parameters

Median Apgar score at 1 min was higher in the Control group but all other perinatal parameters, including birthweight, sex, prolonged pre-mature rupture of membranes, administration of pre-natal steroids, and mode of delivery were similar between the groups (Table 1).

### Ventilatory Parameters

Infants in the Ligated group needed ventilatory assistance for a longer period, with mainly prolonged invasive ventilation (Table 1). Infants in the Ligated group also received more post-natal steroids. On the day before surgery, 35 infants (80%) were on invasive ventilatory support and 9 (20%) were on CPAP.

### Inotropic Support and Transfusions

There was no difference in inotropic support between the groups (Table 1). Infants in the Ligated group received in average two red blood cell transfusions more than infants in the Control group during their hospital stay, but no infants received transfusion in connection to their surgery for PDA.

### Pharmacological Treatment of PDA

The same amount of infants received pharmacological treatment in both groups and during the same time interval (Table 1). One infant in the Control group received treatment with indomethacin, whereas all other treated infants received ibuprofen according to the described protocol. Five infants in the Ligated group and seven infants in the Control group received acetaminophen as analgesic (20 mg/kg rectally twice daily for 2–5 days) in connection with surgery for NEC (*p* = 0.757). No other infants received acetaminophen before discharge.

All infants in the Ligated group had hsPDA during the first week of life, compared to 31 infants (70%) in the Control group (*p* < 0.001). Eleven infants in the Control group had a closed ductus arteriosus at the first echocardiographic examination and none in the Ligated group (Table 2). All infants were examined at the same time after birth.

**Table 2:** Echocardiography before and after pharmacological treatment.

	**Ligated**	**Control**	
	***n* = 44**	***n* = 44**	***p***
**Pre-treatment**			
Timing, day	2 (1–3)	2 (1–2)	0.169
Ductus open, *n*	43 (98%)	33 (75%)	0.004
Ductus diameter, mm	1.9 ± 0.7	1.5 ± 0.9	0.022
Ductus Vmax, m/s	1.32 ± 0.42	1.38 ± 0.51	0.640
LA/Ao	1.8 ± 0.4	1.6 ± 0.4	0.213
hsPDA, *n*	35 (80%)	24 (55%)	0.023
**Post-pharmacological treatment**	***n*** **= 23**	***n*** **= 19**	
Timing, day	14 (9-21)	13 (6-30)	0.795
Ductus open, *n*	22 (96%)	11 (58%)	0.006
Ductus diameter, mm	2.0 ± 0.8	0.9 ± 0.9	0.001
Ductus diameter change, mm	0.0 ± 1.1	−0.9 ± 1.2	0.043
Ductus Vmax, m/s	1.32 ± 0.38	1.66 ± 0.57	0.074
LA/Ao	1.7 ± 0.5	1.6 ± 0.4	0.647
hsPDA, *n*	12 (52%)	5 (26%)	0.120

*Continuous variables are presented as mean ± standard deviation or median and interquartile range (IQR), and categorical variables are presented as number and percent. Vmax, maximal flow velocity; LA/Ao, Left atrium to aortic root ratio; hsPDA, hemodynamically significant patent ductus arteriosus*.

All 23 of the pharmacologically treated infants in the Ligated group underwent post-treatment echocardiography, while 19 of 22 (86%) of the treated infants in the Control group were examined (Table 2). The success of pharmacological treatment was most limited in the Ligated group where all but one infant had an open PDA after treatment, in comparison to 11 infants in the Control group. The ductal diameter was unchanged in the Ligated group, whereas it was reduced after treatment in the Control group (Table 2).

### Multivariate Logistical Regression for Surgical Predictive Variables

In a logistical regression analysis of factors associated with persistent PDA (post-natal steroids, transfusions, pharmacological PDA treatment, and ventilatory days) and echocardiographic findings on the first examination, prolonged need for invasive ventilation was the only factor independently associated with surgical ligation (Table 3).

**Table 3:** Multivariate logistical regression for surgical predictive variables.

	**OR (95% CI)**	***p***
Ductus open at first echo	3.123 (0.088–111.517)	0.531
Ductus diameter at first echo	1.654 (0.521–5.250)	0.393
Pharmacological treatment	1.056 (0.298–3.751)	0.932
Post-natal steroid treatment	–	1.000
Number of transfusions	1.051 (0.842–1.312)	0.661
Days with invasive ventilation	1.041 (1.003–1.079)	0.018

*OR, Odds ratio*.

### Outcomes

Infants in the Ligated group had a higher incidence of severe BPD, whereas mild and moderate BPD were similar to the Control group. The incidence of IVH, NEC, sepsis, and ROP were the same in both groups and there was no difference in mortality (Table 4).

**Table 4:** Outcomes.

	**Ligated**	**Control**	
	***n* = 44**	***n* = 44**	***p***
Mild BPD, *n*	1 (2%)	2 (5%)	1.000
Moderate BPD, *n*	4 (9%)	7 (16%)	0.521
Severe BPD, *n*	38 (86%)	28 (64%)	0.025
IVH grade I–II, *n*	11 (25%)	5 (11%)	0.166
IVH grade III–IV, *n*	5 (11%)	2 (5%)	0.434
ROP stage III, *n*	14 (32%)	15 (34%)	0.653
NEC stage IIIa–IIIb, *n*	5 (11%)	8 (18%)	0.549
Sepsis, *n*	19 (43%)	21 (48%)	0.831
Mortality, *n*	5 (11%)	5 (11%)	1.000
Age at death, day	117 ± 72	191 ± 178	0.417

*Continuous variables are presented as mean ± standard deviation and categorical variables are presented as number and percent. BPD, Bronchopulmonary dysplasia; IVH, Intraventricular hemorrhage; ROP, Retinopathy of pre-maturity; NEC, Necrotizing enterocolitis*.

## Discussion

This retrospective study investigates indications for surgical treatment of PDA and compares outcome for infants subjected to ligation with non-surgically treated controls matched for gestational age and time of birth in order to exclude maturational aspects and other treatment strategies that might have affected our data. The cohort was well-matched for GA and the groups did not differ in mean birthweight, sex balance and proportion of pharmacologically treated infants.

Only 9.5% of all infants born at 22–27 weeks gestational age during the studied period were subjected to surgical ligation. Compared to the rate of surgery in cohorts in previous studies, this reflects a conservative approach in line with the general trend during the last decade, where increased survival has been observed with this strategy but also with a trend toward increased BPD, periventricular leukomalacia and ROP (7, 23). In a recent mini-review of conservative treatment for PDA, the authors stated that there do exist evidence for this approach even in the most severe forms of hsPDA and in extremely pre-term infants without a concomitant increase in either mortality or morbidity (24, 25). In addition, more than half of the infants in the Ligated groups in our study had been primarily pharmacologically treated for PDA and did not show any response to this treatment, reflecting the poor effectiveness of pharmacological treatment in extremely pre-term infants, as previously described by our group and others (26–30).

An early large ductal diameter was associated with future need for surgical ligation in this cohort. This is expected, as a large ductal diameter is one of the criteria for hemodynamical significant PDA used at most centers, including ours, and thus part of the indication for both pharmacological treatment and eventual surgical ligation. The decrease in diameter in infants in the Control group but not in the Ligated group during pharmacological treatment confirms the finding of Pees et al. that a reduction in ductal diameter indicates treatment response (31). There was also a trend toward a lower ductal Vmax being associated with surgical ligation, which is in line with previous results from our center (27, 32).

Early identification of infants with a high risk of developing PDA with considerable hemodynamic effects and with a low chance of successful pharmacological closure is likely to minimize the risk for complications and improve outcomes of surgical treatment. Early low ductal flow velocity and lack of reduction in ductal diameter after failed pharmacological treatment might therefore be useful indices for considering future surgical ligation. Prospective studies of these echocardiographic markers and surgical treatment in this subgroup are thus called for.

In these matched cohorts of extremely pre-term infants, differences in outcomes between the Ligated group and the Control group were mainly related to pulmonary complications. Infants in the Ligated group had more days with invasive ventilation, which was also the only factor independently associated with surgical ligation. As failure to come off the ventilator is an important indication for surgical treatment, this could partly be a confounding effect as indicated by a large study by Weisz et al. (11). In a prospective randomized controlled study of infants born <28 weeks' GA (the PDA-TOLERATE Trial Study) there seemed to be an association between maintained intubation beyond 10 days of life and moderate to severe BPD, when moderate to large PDA persisted more than 10 days and deaths were excluded (33). However, in a prospective multicenter study of infants born between 24^+0^ and 28^+6^ weeks' GA (the INTERPDA Study), a possible reduction of PDA exposure by early medical treatment did not reduce either surgical ligation or BPD incidence, but on the contrary, was associated with a higher mortality (34). Even though the total amount of infants included in our study was smaller in view of the matched cohort study characteristic, the Ligated group was larger and had a lower mean GA than in the two cited studies (*n* = 44/88 vs. *n* = 22/202 and *n* = 37/444; mean GA 24 + 5 weeks vs. 25 + 6 and 26 + 3 weeks; matched cohort vs. PDA-TOLERATE and INTERPDA, respectively), implying that maturational and ventilatory aspects could have interfered with the outcomes of our study while still being relevant for this conservatively managed cohort of extremely pre-term infants (33, 34).

Infants in the Ligated group received more red blood cell transfusions than the Control group. The need for red blood cell transfusions has previously been associated with presence of PDA (35), however, in a study by Sellmer et al. there was no difference between infants with small and large PDAs (36). Lower hemoglobin values have also been associated with BPD and the need for transfusions could therefore be a confounder for pulmonary disease (35–37).

There were no other significant differences in morbidity nor mortality between the groups, highlighting the fact that extremely low gestational age and presence of PDA do not necessarily lead to future surgical treatment. Besides the observed longer duration of mechanical ventilation in the Ligated group, surgical ligation did not have any detrimental effects in this cohort and could be considered a valid alternative in the most pre-term infants with considerable hemodynamic effects of their PDA and low chance of spontaneous or pharmacological closure. Nonetheless, the results of this study need to be interpreted cautiously due to its retrospective nature. The lack of randomization also means that it cannot be ruled out that unidentified clinical factors could have influenced the course of PDA and outcomes after surgical ligation.

## Conclusion

The efficacy of pharmacological treatment was limited in this cohort of extremely pre-term infants. Early large ductal diameter and lack of decrease in ductal diameter during pharmacological PDA treatment predicted future need for surgical ligation. Infants who were surgically treated for PDA needed more respiratory support and had a higher incidence of severe BPD than infants that were not surgically ligated. The compromised pulmonary function might reflect a more pronounced hsPDA and an indicator for surgery in these infants, which was neither associated with increased mortality or other morbidities. With no difference in mortality between our matched surgically and non-surgically treated groups, there is still a need for prospective studies validating the indications for and timing of surgical treatment in extremely pre-term infants, as well as randomized trials to confirm the outcomes of ligation.

## Data Availability Statement

The datasets presented in this article are not readily available because they are only available after ethical approval. Requests to access the datasets should be directed to Swedish Neonatal Quality Register (www.snq.se).

## Ethics Statement

The studies involving human participants were reviewed and approved by the Regional Ethical Review Board. Written informed consent from the participants' legal guardian/next of kin was not required to participate in this study in accordance with the national legislation and the institutional requirements.

## Author Contributions

KO conceptualized and designed the study, supervised data collection, and drafted the initial manuscript. SY conceptualized and designed the study, carried out the data collection, and reviewed the manuscript. MK and RS conceptualized and designed the study, coordinated and supervised data collection, and reviewed and revised the manuscript. RR conceptualized and designed the study and reviewed and revised the manuscript. All authors approved the final manuscript as submitted and agree to be accountable for all aspects of the work.

## Conflict of Interest

The authors declare that the research was conducted in the absence of any commercial or financial relationships that could be construed as a potential conflict of interest.
